# Effectiveness of Simulation-Based Training of Undergraduate Medical Students Regarding the Management of Eclampsia: A Randomized Controlled Educational Trial

**DOI:** 10.7759/cureus.58898

**Published:** 2024-04-24

**Authors:** Mishu Mangla, Naina Kumar, Aparna Jarathi, Nabnita Patnaik, Lalita B Nimmala, Subhrajyoti Roy, Deepak Singla

**Affiliations:** 1 Obstetrics and Gynecology, All India Institute of Medical Sciences, Hyderabad, IND; 2 Anesthesiology and Critical Care, All India Institute of Medical Sciences, Rishikesh, IND

**Keywords:** conventional teaching, teaching and learning in medicine, undergraduate medical student, obstetric emergencies, eclampsia, simulation, medical education

## Abstract

Introduction

Obstetric emergencies, like eclampsia, need a quick and accurate response from the treating physician coming into first contact with the patient. Therefore, all doctors, even primary care physicians, interns, and resident doctors, need training to handle such cases proficiently, leaving minimal chances of error. Providing training for the management of these critical conditions on actual patients is not practically feasible. Clinical simulation in obstetrics can be used for the improvement of these skills for undergraduate and postgraduate students. We conducted a non-blinded randomized controlled trial with the primary aim of developing and implementing a module for training undergraduate medical students on the assessment and management of eclampsia and to evaluate and compare it with traditional didactic lectures or case-based learning.

Methods

The present randomized controlled educational trial was conducted in the Department of Obstetrics and Gynecology, All India Institute of Medical Sciences, Bibinagar, Hyderabad, India. The undergraduate medical students (Phase 3, Part 1) posted in the department during their clinical postings or tutorials were randomized into two groups. A total of 62 students were randomly divided into two groups, Group A and Group B, each consisting of 31 students. However, only 24/31 (77.42%) in Group A and 19/31 (61.3%) in Group B finally agreed to participate in the study. One group (Group A, with 24 participants) was taught the diagnosis and management of antepartum eclampsia through simulation-based training, and the other group (Group B, with 19 participants) was taught the same topic through conventional teaching, which consisted of didactic lectures through PowerPoint presentations and case-based discussion. Learning objectives were kept identical for both groups. Pre- and post-test scores were compared for both groups.

Results

The mean pre-test score of the simulation group was 6.13 ± 1.39, and that of the conventional teaching group was 6.05 ± 1.54. The post-test score of the simulation group was 9.17 ± 1.34, and that of the conventional teaching group was 7.37 ± 1.70. The simulation group showed an extremely significant (two-tailed p < 0.0001) improvement in their post-test scores when compared to their scores before the module was taught. The difference in the scores of simulated teaching (Group A) and conventional teaching (Group B) was also statistically significant (p = 0.005). Simulation-based learning was found to be more interactive, helpful in providing real-life-like experiences, led to better retention and understanding, and motivated the students for self-directed learning.

Conclusion

Although both conventional and simulation-based teaching were useful, simulation-based training was more effective in teaching undergraduate medical students regarding the diagnosis and management of eclampsia. Simulation-based learning is more interactive, provides real-life-like experiences, leads to better retention and understanding, and motivates the students for self-directed learning.

## Introduction

India is a signatory to the United Nations Sustainable Development Goals, which adopted a global maternal mortality ratio (MMR) target of fewer than 70 deaths per 100,000 live births by 2030 [1]. As per the Special Bulletin on MMR released by the Registrar General of India, the MMR of India presently stands at 97/lakh live births. The leading causes of maternal deaths in India are obstetric hemorrhage, pregnancy-related infection (12%), and hypertensive disorders of pregnancy (7%) [2]. Among the hypertensive disorders of pregnancy, eclampsia contributes to the majority of morbidity and mortality [3]. Undergraduate medical students, especially in the final year and internship, may, directly or under supervision, be involved in the care of women during delivery and childbirth. They need to be well equipped and informed about handling basic and common obstetric emergencies, as they may sometimes be the first point of doctor-patient contact. They, too, like health care professionals, have the right to continuous training and efficiency in handling emergency conditions in obstetrics.

Obstetric emergencies, like eclampsia, need a quick and accurate response from the treating physician coming into first contact with the patient. Therefore, all doctors, even primary care physicians, interns, and resident doctors, need training to handle such cases proficiently, leaving minimal chances of error. Providing training for the management of these critical conditions on actual patients is not practically feasible. Clinical simulation in obstetrics can be used for the improvement of these skills for undergraduate and postgraduate students [4]. Simulation has multiple advantages, including the possibility of repeating and practicing situations that, although not very frequent, are crucial in real life. It allows the medical practitioner an opportunity to correct one’s own errors, sometimes by observing the video recording of one’s action and seeing oneself improving over time. However, one of the most important things that have to be kept in mind while designing the simulation scenario is that it must be ethical and involve treating the woman and her attendants, just like that in real life. It should involve all the basic and essential steps required to manage the condition of interest.

Research has not conclusively demonstrated whether simulation-based training is superior to the traditional teaching methods of didactics or the more recently utilized problem-based learning format [4,5]. We therefore conducted a non-blinded randomized controlled trial to determine whether simulation-based training is superior to traditional didactic lecture or case-based learning in the assessment and management of antepartum eclampsia. The primary aim was to develop and implement a module for training undergraduate medical students in the assessment and management of eclampsia and to evaluate and compare it with traditional didactic lectures or case-based learning. A secondary objective was to elicit feedback for the facilitation of the revision of the module and assessment methods.

## Materials and methods

Study setting, participants, and randomization

**Figure 1:**
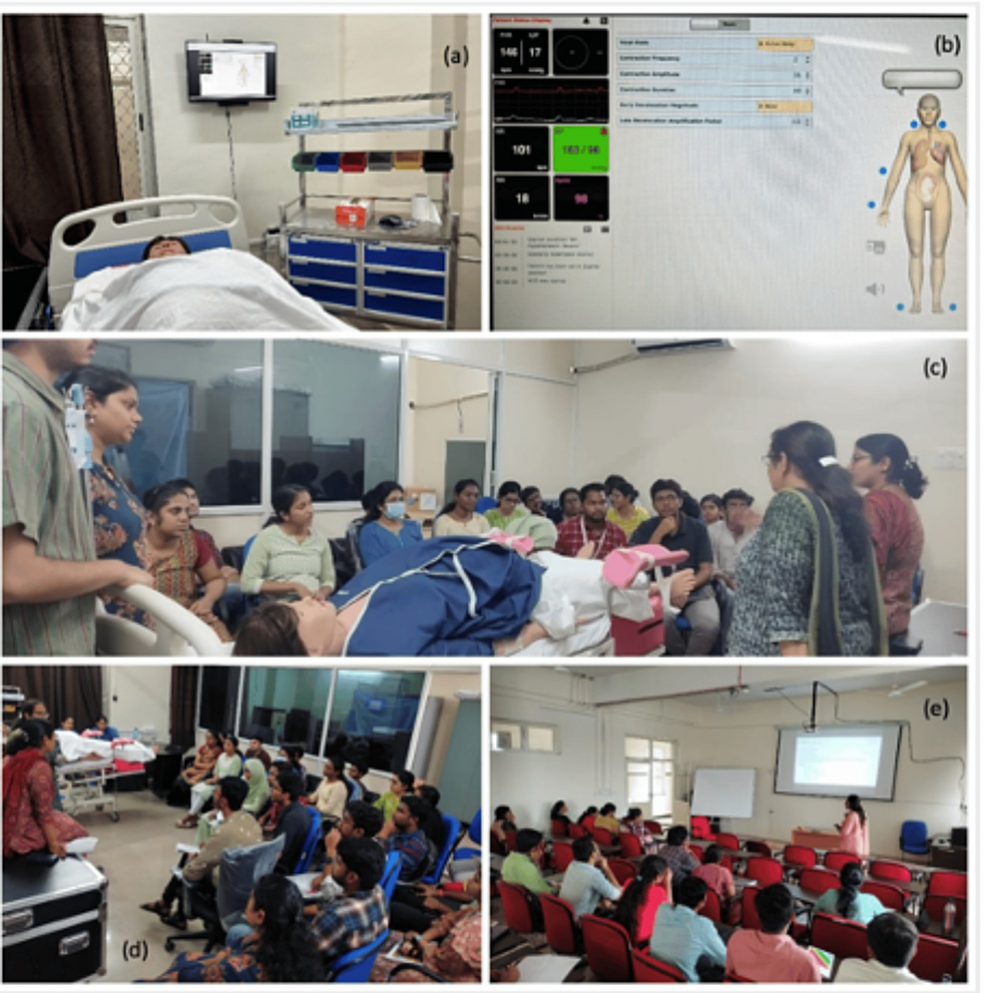
Implementation of (a-d) a simulation-based training module for students in one group and (e) a conventional didactic lecture for the other group

The present randomized controlled educational trial was conducted in the Department of Obstetrics and Gynecology, All India Institute of Medical Sciences, Bibinagar, Hyderabad, India, after obtaining due permission from the institutional medical education cell and ethics committee. The department is equipped with a high-fidelity birthing simulator representing a full-term pregnant adult woman, which responds to clinical intervention, instructor control, and pre-programmed scenarios and allows for the observation of both maternal and fetal vital signs. The study population consisted of undergraduate medical students of the institute in Phase 3 of MBBS (Part 1) without any exclusion criteria. The students posted in the department during their clinical postings or tutorials were randomized into two groups, Group A and Group B, based on a random number table. Group A was taught the diagnosis and management of antepartum eclampsia through simulation-based training, and Group B was taught the same topic through conventional teaching, which consisted of didactic lectures through PowerPoint presentations and case-based discussion (Figure 1).

**Figure 2:**
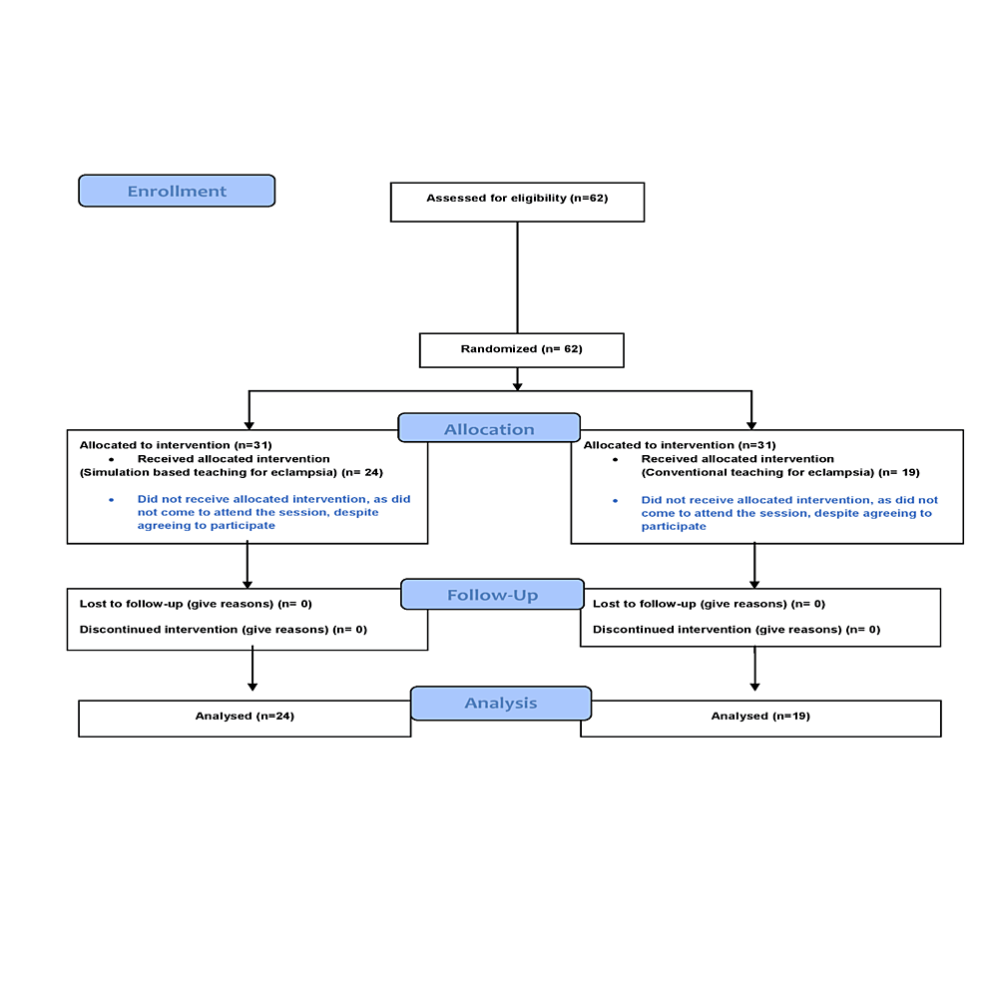
CONSORT flow diagram of the students enrolled in the present study CONSORT, Consolidated Standards of Reporting Trials

The Consolidated Standards of Reporting Trials (CONSORT) flow diagram of the study population is shown in Figure 2.

The duration of both sessions was three hours. The use of the simulator was restricted to the study, and the results of the study did not affect their internal assessment. Learning objectives were kept identical for students receiving both conventional teaching and simulation-based training for the topic.

The study was planned and implemented in four phases, as described below.

Phase 1: Curriculum development, checklists, and scoring for a simulation-based module on eclampsia

**Figure 3:**
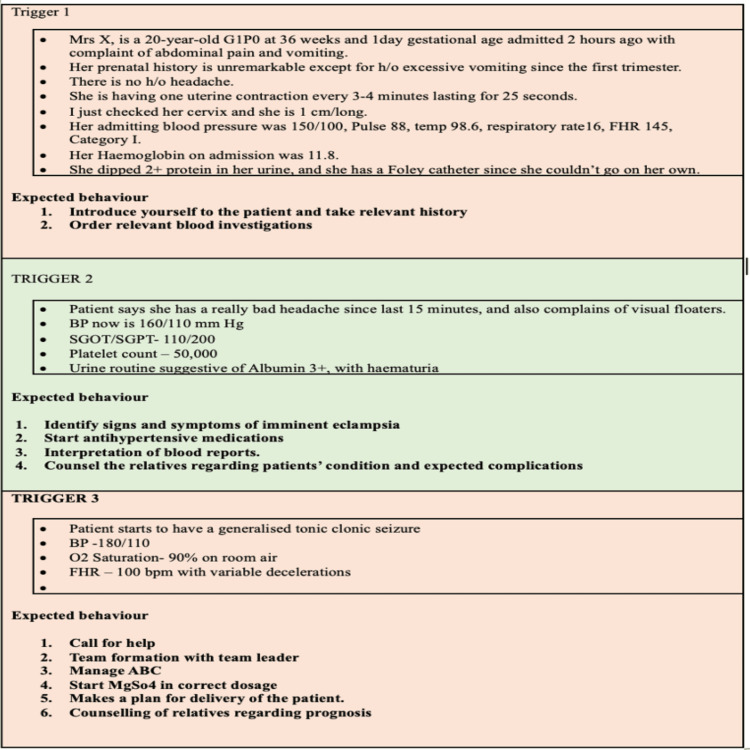
Case scenario on antepartum eclampsia utilized for simulation-based teaching in the present study

The module was designed by the faculty of the Department of Obstetrics and Gynecology, taking into consideration the competencies that an undergraduate medical student is expected to acquire during the MBBS course. A pre-test and post-test design was utilized, in which the students were evaluated on their abilities and competencies before and after the simulation intervention and before and after conventional teaching. The students in both groups were enrolled only after ensuring that the relevant topics had been covered in their course to ensure that they had the basic knowledge regarding eclampsia. The course was divided into two 90-minute sessions, each aimed at teaching the diagnosis and management of eclampsia. Prior to the beginning of the module, a 10- to 15-minute oral presentation was made to explain the educational objectives, followed by a 10-minute video to precisely recall the basic definitions related to the above conditions and remind them of the basic anatomical and physiological concepts related to hypertension in pregnancy. For simulation-based teaching, case scenarios of eclampsia were developed by the principal and co-investigators and finalized after review by the medical education unit of the institute (Figure 3).

Phase 2: Training of the educators and nursing officers who helped in the creation of real-life scenarios in the simulated and controlled conditions

The educators and trainers for the above course were senior obstetricians. Each of the trainers was provided with a detailed instruction manual describing how to use the mannequins, the educational objectives of the course, and the duration of each step of the course so as to maintain uniformity. In addition, the principal investigator coordinated the education program and demonstrated the operation of the simulation equipment to all educators before the course. The objective of each session was to help the students identify the condition of interest (eclampsia) and provide primary care management, as is expected from a primary care physician or doctor posted in the emergency department.

Phase 3: Implementation of the training program for undergraduate students

The program was implemented over the course of one month through tutorial sessions and clinical postings. As discussed in the methodology, one group will be taught through simulation-based learning and the other through conventional teaching methods.

Phase 4: Assessment

At the end of the session, each student was individually exposed to various case scenarios related to eclampsia using standardized patients, and their performance was assessed with the help of a checklist that was developed as a part of the study (Table 1).

**Table 1:** Checklist used for the evaluation of both groups

Action expected from the student (weightage)
Greets and introduces oneself to the patient (0.5)
Elicits a complete history from the patient (1)
Identifies the warning signs of preeclampsia or the imminent signs of eclampsia (2)
Counsels the relatives regarding fetomaternal prognosis from time to time (1)
Calls for help (1)
Facilitates team building (1)
Handles emergencies in a coordinated manner with a team with designated roles for each member (1)
Ensures airway, breathing, and circulation management (2)
Prepares 10 ampoules (20 ml = 10 g) of 50% MgSO4 (2)
Prepares two syringes (10 ml syringe and 22 gauze needles) with 5 g of 50% magnesium sulfate solution (1)
Cleans the injection site carefully with an alcohol wipe (0.5)
Administers 5 g by deep IM injection in one buttock (0.5)
Clean the injection site carefully in the alternate buttock with an alcohol wipe (0.5)
Administers 5 g by deep IM injection into the other buttock (0.5)
Disposes of the used needle and syringe in a puncture-proof box (0.5)
Decides to administer antihypertensive medication (2)
Administers the required dose of the antihypertensive agent correctly (1)
Plans for delivery (2)

The method of assessment was common for both groups. A checklist regarding critical actions required to manage the condition of interest was developed, and the individual actions were weighted by consensus, with higher point values assigned to actions deemed more critical. Components of the checklist included relevant history, physical examination, diagnostic evaluation, and treatment. Each item was scored in a performed or not performed fashion and also in what order they were performed. A note was also made regarding interpersonal communication and team-building efforts. Each student’s performance was evaluated in real time during the simulated scenario. Each of these case scenarios was developed by experienced obstetricians, taking into consideration the basic competencies expected to be fulfilled by undergraduate medical students.

Phase 5: Feedback

The students were requested to provide feedback regarding the module, highlighting both the most and least useful aspects of the course and making suggestions for improvement. A free response area was provided so that they could pen down their thoughts regarding the module.

Statistical analysis

The data collected were entered into an Excel file, and statistical analysis was done using GraphPad InStat software (GraphPad Software Inc., La Jolla, California, United States). Continuous variables have been expressed as mean ± standard deviation or numbers (percentages). The Kruskal-Wallis test was used to study the normal distribution of both groups. For continuous variables that are not normally distributed, the Mann-Whitney test was used for comparison between groups. The χ2 test was used for comparison between categorical variables. A p-value < 0.05 was considered statistically significant. The free-response areas of the feedback form were independently analyzed by two independent observers, and opinions representing more than 20% of the study population have been reported.

## Results

A total of 62 students were randomly divided into two groups, Group A and Group B, each consisting of 31 students. However, only 24/31 (77.42%) in Group A and 19/31 (61.3%) in Group B finally agreed to participate in the study. Therefore, Group A had 24 and Group B had 19 participants. The baseline characteristics of the study participants are presented in Table 2.

**Table 2:** Baseline characteristics of the study participants

Characteristics of the study population	Group A, Simulation-based teaching (n = 24)	Group B, Conventional teaching (n = 19)
Mean age (years)	22.18 ± 1.46	21.89 ± 1.20
Gender		
Male	14	10
Female	10	9
Preferred learning style (VARK model)		
Visual	7	7
Auditory	8	6
Reading and writing	7	5
Kinaesthetic	2	1
Handedness		
Right-handed	21	17
Left-handed	3	1
Mixed/ambidextrous	0	1
Pretest scores (baseline knowledge regarding management of eclampsia)	6.13 ± 1.39	6.05 ± 1.54

**Figure 4:**
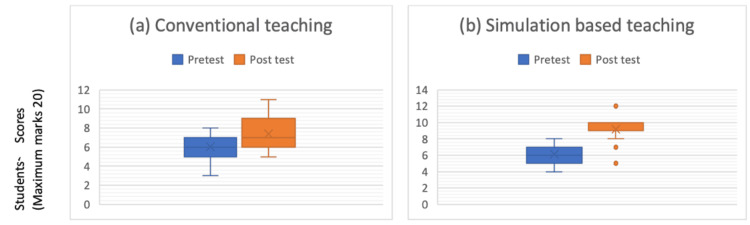
Distribution of the scores in the (a) conventional teaching group and in the (b) simulation-based teaching group

The mean pretest score of the simulation group was 6.13 ± 1.39, and that of the conventional teaching group was 6.05 ± 1.54. The post-test score of the simulation group was 9.17 ± 1.34, and that of the conventional teaching group was 7.37 ± 1.70. The median, quartiles, and distribution of scores for both groups are represented in Figure 4.

**Figure 5:**
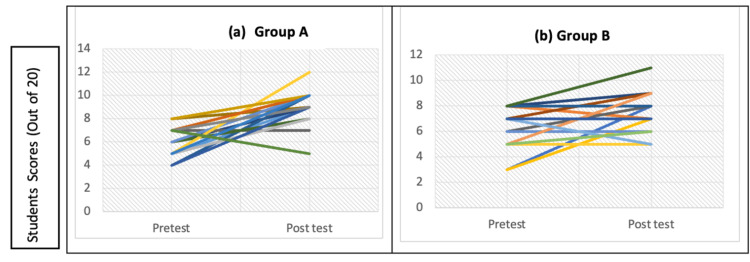
Performance of individual students in (a) Group A (simulated teaching) and (b) Group B (conventional teaching)

The individual scores of students in both groups are graphically represented in Figure 5.

The simulation group showed an extremely significant (two-tailed p < 0.0001) improvement in their post-test scores when compared to their scores before the module was taught. Although the improvement in the scores of students who were taught eclampsia through conventional teaching was also significant (two-tailed p < 0.016), it was less as compared to the simulation group. The difference in the scores of simulated teaching (Group A) and conventional teaching (Group B) was also statistically significant (p = 0.005). Students’ feedback and their experiences are summarized in Table 3.

**Table 3:** Students’ experiences with the session

Themes	Related verbatims
Better understanding	“Very enjoyable and effective session. Understood a lot through this session. Please keep arranging more such sessions. Firstly, thank you so much for all your efforts, ma’am! It was a really informative and interactive session. This makes us memorize things easily. I look forward to more sessions like this.”
Better retention	“It was amazing to see the management of an emergency situation live, and it would help me retain information for a longer time. Was very interesting and innovative. We never felt bored or lost our presence of mind. Now I remember more than myself attending a class with PPT. It was a really nice session, and the visualization of the real scenario was very helpful. Could understand more clearly, as it was shown that it will stay in the mind for a long time. Thank you. This was the best learning session in obstetrics that I ever had! Please take more such sessions for the management of emergency conditions. This type of teaching will stick with us more than PPT learning! Thank you so much for this session!”
Interactive with no loss of interest	“It was so interactive as the focus was on a smaller number of students, and there was more scope for one-on-one interaction, active participation of students, and doubt clarification. Live demonstration helps us understand the importance of the topic and the execution of proper management measures in real-life situations.”
Gives the experience of a real-life situation	“It was so interactive as the focus was on a smaller number of students, and there was more scope for one-on-one interaction, active participation of students, and doubt clarification. Live demonstration helps us understand the importance of the topic and the execution of proper management measures in real-life situations. The session was more practical based, and such a session will be useful for the students in the future if they face the same situation. They will understand how to manage emergency cases.”
Systematic	“It was a good session that followed the topic in a systematic manner. The combination of the two lectures helped solidify concepts.”
More practical	“It was very useful, as we see in the scenario. We can never forget what should be done in such cases, rather than theoretical teaching where we can get confused and not have this clarity.”
Left the students yearning to learn and do more	“We have the best visual learning method. Thanks for the new method of teaching. I wish to have hands-on management skills like how to place a mouth guard, catheter insertion, and the preparation of magnesium sulfate solution. It was a great session, and I hope to have it for postpartum hemorrhage too. Please cover all must-know topics in this manner. The session is really beneficial for us. We want to see everything in detail, like how to put a urinary catheter in and mix the solution of MgSO4.”

Simulation-based learning was found to be more interactive, helpful in providing real-life-like experiences, led to better retention and understanding, and motivated the students for self-directed learning.

## Discussion

The management of obstetric emergencies like postpartum hemorrhage, eclampsia, uterine inversion, cord prolapse, and fetal distress in labor requires a quick and accurate response from the primary care provider, be it a consultant, resident doctor, intern, or a primary care physician posted at a primary health center or a tertiary care hospital. All the actions or the basic measures required to manage such conditions should become reflexes, right from the time they pass MBBS and join an internship. The conventional method used for teaching the management of all such conditions has been didactic lectures, case-based discussions, or demonstrating on real patients for some students who are lucky enough to come across such patients at the time that they are posted in the department. It is not possible for all the students in any batch to be able to see and learn the management of such conditions in real life during their postings in the department. Hence, there is a need to expose all the students to such critical scenarios either in real life or, if not possible, using real-time simulators, where they can be made to encounter these situations, the stress and sense of responsibility toward the patient, and how to handle such cases [5].

Simulators were initially used for training pilots way back in the 1920s, as their job requires high precision, and even a small fault on their part can be life-threatening for them or their passengers. The use of simulators in medical training started in 1960 and included models, mannequins, and even standardized patients. Simulation is an effective way for residents and students to develop their skills in a safe learning environment. Simulation offers a realistic approach to practicing such skills without the potential to cause harm to a living patient. Teaching in a simulated environment has an additional advantage in that one clinical condition can be recreated multiple times so that all the students get a chance to witness such a situation, and they can practice again and again till they attain the desired perfection to manage the condition of interest. However, when designing a simulation-based module, the target audience should be kept in mind because simulation can have varying levels of difficulty [6]. Therefore, before implementing the module for teaching eclampsia to undergraduate medical students, we clearly defined the learning objectives first. While preparing the checklist for assessment, due care was taken to ensure that only the actions expected of an undergraduate medical student were included. The clinical case (training module) and the checklist prepared can be a useful guide for anyone planning to conduct similar sessions for teaching.

We found that both methods of teaching, i.e., simulation-based teaching as well as conventional teaching through didactic lectures and case-based teaching, were effective in teaching the students about the diagnosis and management of eclampsia. In both groups, the post-test scores of the students were significantly better than their pre-test scores. However, the difference in the post-test scores between simulated teaching (Group A) and conventional teaching (Group B) was also statistically significant (p = 0.005), favoring simulation as a better and more effective method of teaching. It is, however, important to note that the learning experiences of the students varied widely in both groups. The student feedback regarding the sessions, as described in Table 3, favored simulation. The students found the simulation session to be more systematic, interesting, and practical. The authors also found that simulation was very effective in arousing the interest of students in doing everything themselves and also left them yearning to learn more. Simulation as a better and more effective method has been found in other studies on midwives [7-10].

Although simulation as a teaching method is not new, it has been in widespread use to teach nursing and midwifery students, resident students, and even in faculty development programs [11-15]. The present study was unique as we used this as the modality to teach undergraduate students, who are mainly taught through lectures and PowerPoint presentations. McCoy et al. reported a randomized trial comparing simulation-based teaching to problem-based learning for undergraduate medical students and found simulation better for the acquisition of critical assessment and management skills [9]. An interprofessional team collaboration between undergraduate midwifery students and undergraduate medical students was recently carried out for teaching obstetric emergencies by Tauscher et al., and they found such types of training sessions are extremely useful in teaching team building, interpersonal communication, and soft skills [10,16].

The present study is one of the very few that has tried to introduce and implement simulation as a method of teaching undergraduate medical students. The study, however, had certain limitations. Firstly, this study reports on the performance of a limited number of medical students studying in a single institution, so caution must be used in extrapolating the results to students and other institutes. Secondly, the study was only partially blinded, and both the participants and their educators were aware of which group they were allotted to, which could have led to bias. Thirdly, results should be analyzed with caution, keeping in mind that at any time, the implementation of a new educational activity, such as role play and simulation in the current situation, is associated with increased enthusiasm from both educators and students and may also have impacted the results.

## Conclusions

Although both conventional and simulation-based teaching were useful, simulation-based training was more effective in teaching undergraduate medical students regarding the diagnosis and management of eclampsia. Simulation-based learning is more interactive, provides real-life experience, and provides real-time feedback, as the students and facilitators can go back to sessions through recorded videos. It also helps in building and understanding team dynamics, improves communication skills, leads to better retention and understanding, and motivates the students for self-directed learning.
